# A Mobile Intervention to Promote Low-Risk Drinking Habits in Young Adults: Protocol for a Randomized Controlled Trial

**DOI:** 10.2196/29750

**Published:** 2021-06-07

**Authors:** Nikolaos Boumparis, Mieke H Schulte, Annet Kleiboer, Anja Huizink, Heleen Riper

**Affiliations:** 1 Department of Clinical, Neuro and Developmental Psychology Amsterdam Public Health Research Institute Vrije Universiteit Amsterdam Amsterdam Netherlands

**Keywords:** alcohol, lifestyle, drinking, young adults, digital, mobile app, COVID-19

## Abstract

**Background:**

Young adults’ drinking habits commonly exceed recommendations for low-risk drinking, which may have a negative effect on their mental, social, and physical health. As smartphones are highly accessible to young adults, mobile apps could be used to support young adults to develop low-risk drinking habits and improve their general health.

**Objective:**

The objective of this study is to evaluate the effectiveness of Boozebuster, a self-guided mobile app based on healthy lifestyle–related components that aim to develop and maintain low-risk drinking habits among young adults.

**Methods:**

This two-arm, parallel-group randomized controlled trial will investigate whether a 6-week self-guided mobile intervention (Boozebuster) targeting drinking behavior is more effective than a minimal intervention consisting of an educational website on alcohol use and its consequences for young adults. We will recruit 506 young adults (aged 18-30 years) from the Netherlands via an open recruitment strategy by using an open access website. All outcomes will be self-assessed through questionnaires. The primary outcome is the quantity and frequency of alcohol consumption in standard drinks (10 g ethanol per standard drink) per month (timeline follow-back [TLFB]). Secondary outcomes include binge-drinking sessions per month, alcohol-related problem severity (Rutgers Alcohol Problem Index), cannabis use frequency and quantity in grams (TLFB), depressive symptoms (Center for Epidemiological Studies Depression Scale), perceived stress (Perceived Stress Scale), engagement (Twente Engagement with eHealth Technologies Scale), readiness to change (Readiness to Change Questionnaire), mental well-being (Warwick-Edinburgh Mental Wellbeing Scale), trauma and COVID-19–related trauma (Short-Form Posttraumatic Stress Disorder Checklist for Diagnostic and Statistical Manual of Mental Disorders, 5th Edition), impulsivity (Urgency, Premeditation, Perseverance, Sensation Seeking, Positive Urgency Impulsive Behavior Scale), study or work performance (Individual Work Performance Questionnaire), and treatment adherence. Baseline (T0), 6-week postbaseline (T1), and 3-month postbaseline (T2) assessments will be conducted and analyzed on the basis of the intention-to-treat principle using multilevel mixed modeling analyses.

**Results:**

Recruitment began in September 2020. We received 933 registrations via our study information website; 506 participants have completed the T0 assessment, 336 participants have completed the T1 assessment, and 308 participants have completed the T2 assessment as of May 2021. The study is still in progress, and results will be reported in 2021 and 2022.

**Conclusions:**

Self-guided mobile interventions based on a lifestyle approach might be an attractive approach for young adults due to their preference on self-reliance, healthy living, and increased perceived anonymity. Such interventions are yet understudied, and it is known that interventions addressing solely problem drinking are less appealing to young adults. We hypothesize that the Boozebuster mobile app will effectively reduce drinking levels compared to an alcohol educational website (control condition). If effective, our intervention could be an inexpensive and scalable public health intervention to improve drinking habits in young adults.

**Trial Registration:**

Netherlands Trial Register NL8828; https://www.trialregister.nl/trial/8828

**International Registered Report Identifier (IRRID):**

DERR1-10.2196/29750

## Introduction

It is well known that the prevalence of alcohol consumption among young adults is high and that their drinking habits commonly exceed low-risk drinking guidelines [1]. A study by the Dutch National Institute for Public Health and the Environment (Rijksinstituut voor Volksgezondheid en Milieu) showed, for example, that 8.9% of young adults were considered problem drinkers, defined as individuals who drink more than 21 standard glasses of alcohol per week for males and more then 14 standard glasses of alcohol per week for females. Alcohol consumption among young adults is characterized by a drinking pattern that differs from the general population. Specifically, we know that young adults are more likely to binge drink, especially during the weekends [2]. In addition, binge drinking occurs in about 11.4%-19.4% of young adults (18-29 years of age) compared to approximately 7.7% of the general population [3]. Consequently, young adults’ drinking patterns pose a significant negative effect on young adults’ mental, social, and physical health [4].

According to the literature, face-to-face interventions delivered in individual or group settings targeting young adults’ drinking patterns are effective [5,6]. However, given that young adults commonly avoid traditional counseling services due to perceived stigma and their preference for self-reliance [7,8], a potential solution to overcome these obstacles might be to provide self-guided digital interventions, which require limited resources compared to traditional prevention and treatment services.

Ample digital interventions that are aimed to reduce drinking levels in young adults, mostly in university settings located in the United States or Australia, have been previously evaluated. The majority of these interventions consist of internet-based single-session interventions, based on personalized normative feedback (PNF), that aim to correct erroneous perceptions of peer drinking levels by assessing personal drinking levels and consequently comparing them with drinking levels in similar-aged peer groups [9]; general alcohol education programs that are commonly incorporated within general education curriculums and provide educational modules about alcohol use, its dangers, and often focus on ensuring students comply with the law [10]; or brief interventions that screen for problematic drinking patterns and present feedback motivating individuals to alter those patterns [11].

The majority of the above-described digital interventions are delivered via a computer or web browser. However, it has been claimed that mobile apps are more suitable for young adults given the flexibility, interactivity, and their spontaneous nature [12]. However, a recent review assessed mobile apps developed with the purpose of managing alcohol consumption found that the evidence is promising but still inconclusive given the mixed results [13].

Although single-session interventions can reach a high number of individuals due to their appealing, limited time requirement, they are associated with a few important disadvantages such as the relatively small effect sizes produced and the limited longevity of those effects. For example, meta-analyses assessing single-session interventions show small effect sizes when compared to assessment only, attention-matched, or active controls ranging from g=0.18 to g=0.29 [5,14-18]. In addition, studies that assess long-term outcomes often show that those effects cease to exist in follow-up periods [19]. Lifestyle medicine is a relatively new approach for the potential prevention, promotion, and management of mental health conditions. It involves the use of environmental, behavioral, and psychological principles to improve physical and mental well-being by modifying lifestyle factors such as diet, physical activity, relaxation, sleep, and stress [20]. Although lifestyle modification has been extensively studied in particular regarding the prevention of chronic diseases [21], research about its value in curbing drinking among young adults is very limited. However, studies have shown that digital lifestyle interventions that target multiple behaviors may increase and prolong the effectiveness of behavioral change interventions targeting young adults [22-24].

Therefore, in our intervention, we decided to incorporate relevant lifestyle components that have been shown to be perceived as relevant and important for our target group’s general well-being [25-27]. Such lifestyle-related topics include sleeping habits, mood, and perceived stress. We expect that by providing young adults with relevant lifestyle components in addition to the alcohol-related intervention content, we have a potential to increase and prolong effect sizes regarding drinking outcomes, while retaining the acceptability and reach of single-session interventions.

## Methods

### Study Design

We will conduct a two-arm, parallel-group randomized controlled trial (RCT) comparing a 6-week mobile app (Boozebuster) with an active educational website control condition. Participants in the Boozebuster condition will receive access to the self-guided mobile app; participants in the control condition will receive access to an educational website containing information about the effects and consequences of alcohol use on health. The Scientific and Ethical Review Committee of the Faculty of Behavioral and Movement Sciences of the Vrije Universiteit Amsterdam has approved the study protocol, informational letter, and informed consent process. The primary and secondary outcomes in addition to the exact timepoints at which each measure will be applied is presented in Table 1. The study is registered with the Netherlands Trial Registry (NL8828).

**Table 1:** Overview of study measurements, timepoints, and instruments.

Outcome measures	Baseline (T0)	6-week postbaseline (T1)	3-month postbaseline (T2)
Sociodemographics	✓		
Alcohol consumption, frequency, and quantity (TLFB^a^)	✓	✓	✓
Binge-drinking frequency	✓	✓	✓
Cannabis consumption, frequency, and quantity (TLFB)	✓	✓	✓
Depressive symptoms (CES-D^b^ Scale)	✓	✓	✓
Perceived stress (PSS^c^)	✓	✓	✓
Alcohol-related problem severity (RAPI^d^)	✓	✓	✓
Readiness to change (RCQ^e^)	✓	✓	
Task performance (IWPQ^f^)	✓	✓	✓
Engagement (TWEETS^g^)	✓	✓	
Mental well-being (WEMWBS^h^)	✓	✓	✓
Short-form PCL-5^i^	✓	✓	✓
Short-form PCL-5 – COVID-19	✓	✓	✓
UPPS-P^j^ Impulsive Behavior Scale	✓	✓	✓

^a^TLFB: timeline follow-back.

^b^CES-D: Center for Epidemiological Studies Depression.

^c^PSS: Perceived Stress Scale.

^d^RAPI: Rutgers Alcohol Problem Index.

^e^RCQ: Readiness to Change Questionnaire.

^f^IWPQ: Individual Work Performance Questionnaire.

^g^TWEETS: Twente Engagement with eHealth Technologies Scale.

^h^WEMWBS: Warwick-Edinburgh Mental Wellbeing Scale.

^i^PCL-5: Posttraumatic Stress Disorder Checklist for Diagnostic and Statistical Manual of Mental Disorders, 5th edition.

^j^UPPS-P: Urgency, Premeditation, Perseverance, Sensation Seeking, Positive Urgency.

### Inclusion Criteria

Young adults will be eligible for the RCT if they meet the following criteria: (1) aged between 18 and 30 years, (2) willingness to develop healthy lifestyle behaviors and low-risk drinking habits, (3) provision of informed consent digitally, (4) proficiency in reading and writing in Dutch, (5) access to an Android or IOS device with connection to the internet, and (6) possession of an email address. We did not apply a minimum drinking severity inclusion criterion. Our reasoning for not screening drinking severity is based on the fact that the Dutch drinking guidelines recommend an alcohol consumption level of zero or no more than 1 alcoholic drink per day.

### Recruitment

We will make use of an open recruitment strategy, specifically, by means of advertisements on social media and health-related websites and via posters on educational facilities. These advertisements contain a link to our study information website [28]. Interested respondents can apply by leaving their email address, and they will be consequently contacted via email by the research team. The participants will be invited to complete a baseline assessment (T0), 6-week postbaseline assessment (T1), and 3-month postbaseline assessment (T2) via the computerized Castor Electronic Data Capture (EDC) system [29]. All participants will be offered an incentive of €10 (US $12.2) for completing the T1 assessment and an additional €10 (US $12.2) incentive for completing the T2 assessment.

### Randomization

**Figure 1:**
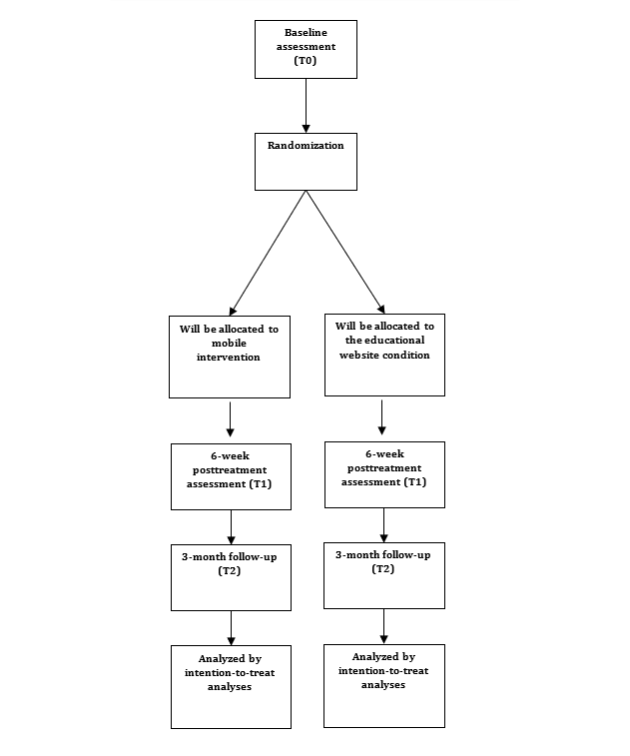
Overview of the participant flow for the randomized controlled trial.

First, participants will be screened, asked to fill out the baseline assessment (T0), and then randomized into either the mobile intervention or the educational website group. The allocation sequence will be automatically generated via the computerized Castor EDC system by using a 1:1 allocation ratio with random block sizes of 4 and 6, which also ensures allocation sequence concealment. Participants will be informed about the outcome of the randomization via email by a member of our research team. Initial stratification will be into two groups according to gender (ie, male and female). Within these two groups, participants will be further stratified into two groups categorized by adherence or nonadherence to the Dutch drinking guidelines. Nonadherence will be defined as the consumption of more than 7 drinks per week or at least one binge-drinking session (≥5 drinks for male participants and ≥4 drinks for female participants) in the past 30 days. This will create four strata. Because of the nature of our study design, blinding of group allocation for participants is not possible. Figure 1 provides an overview of the trial flow.

### Sample Size

Previous meta-analyses assessing drinking reduction in young adults compared to assessment only, attention-matched, or active controls [17,18] found small effect sizes of about g=0.25. Although we expect the Boozebuster intervention to outperform previous interventions in reducing the quantity and frequency of alcohol consumption in comparison to the control condition at the posttest assessments, we decided to base our sample size calculation on this conservative effect estimate. These estimations would result with α=.05 and 1–β=.80 in a sample size of 253 per study arm (ie, N=506).

### Intervention

Boozebuster is a mobile app aimed at developing low-risk drinking habits in young adults according to the Dutch drinking guidelines. It has been developed by Vrije Universiteit Amsterdam in collaboration with the Institute for Systems and Computer Engineering, Technology and Science [30]. Boozebuster is based on the Moodbuster platform, which is being used for a variety of research projects funded by the European Union [31,32].

Boozebuster focuses on the importance of the individuals’ motivation, self-efficacy, and environmental constraints with regard to drinking. The behavioral change techniques used include PNF [18], motivational interviewing [33], cognitive behavioral therapy [34], goal setting [35], self-monitoring [36], protective behavioral strategies [37], and mindfulness [38].

Boozebuster contains a total of seven modules and was developed with the following rationale in mind: the PNF module serves as the first assessment of young adults’ drinking patterns and compares it with peer norms and the official Dutch drinking guidelines [39]. This procedure creates awareness among the individuals about their alcohol consumption in relation to their peers and has shown to be an effective approach in initiating drinking-related changes [40]. The incorporated motivational interviewing module motivates the individuals to change their alcohol-related behaviors by assisting them in building confidence in their abilities for achieving their goals. In addition, Boozebuster includes a variety of lifestyle support modules, such as relaxation, improving sleep, drinking diary, gratitude diary, and an emergency button specifically designed to provide recommendations for acute cravings. These modules provide individuals with tools to deal with cravings, peer pressure, and stress that might otherwise interfere with their drinking-related goals.

Participants in the Boozebuster group will be able to monitor their daily alcohol consumption via ecological momentary assessments (EMA) [41] in terms of the amount of standard drinks consumed, their mood via daily mood ratings on a scale of 1 (low) to 10 (high), and their sleep quality via daily sleep quality ratings on a scale of 1 (low) to 10 (high). Participants will also be able to monitor their progress on the three abovementioned key behaviors (ie, drinking, mood, and sleep) via visual feedback (ie, visualization of their scores) through the mobile app.

**Figure 2:**
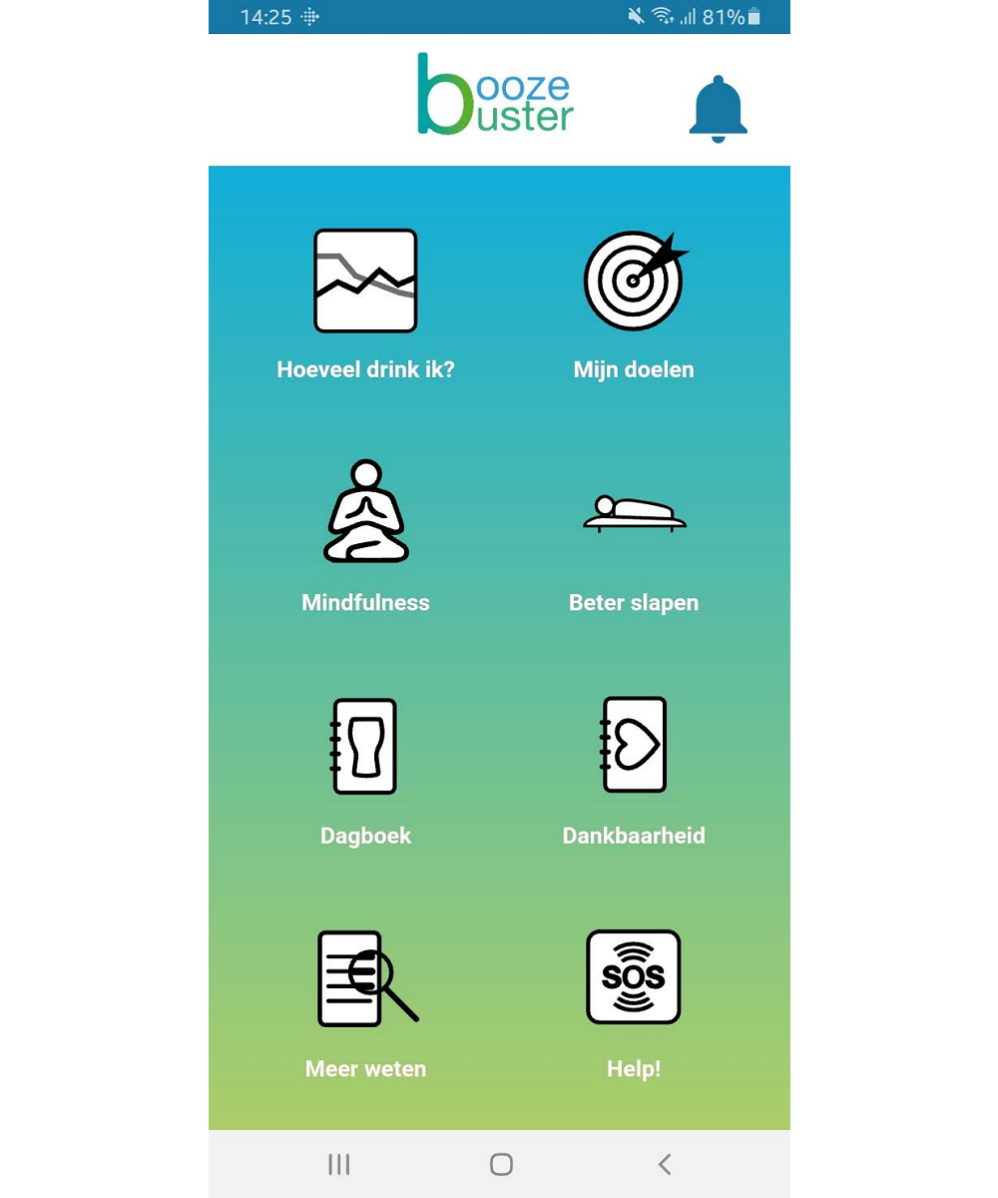
Screenshot of the Boozebuster mobile app (home screen).

Participants are free to use Boozebuster during the 6-week study duration as frequently as desired. The reason we opted for a 6-week period is because it is the recommended timeframe in which alcohol-related changes can occur and be measured [42]. Hence, participants will be stimulated and motivated to engage with our intervention via the daily reminders of the EMA function during the 6 weeks of study participation. Figure 2 provides an overview of the Boozebuster mobile app and its modules.

### Educational Website Condition

Participants in the control condition will receive access to an educational website containing information on the effects and consequences of alcohol use on health. Only participants allocated to the control condition will have access to this website.

### Outcome Measures

Given that applied outcome measures in the alcohol intervention literature vary considerably we decided to follow the advice and included key outcomes based on the recommendation of the Outcome Reporting in Brief Intervention Trials on Alcohol (ORBITAL) framework [43].

#### Primary Outcomes

##### Alcohol Consumption, Frequency, and Quantity

The primary outcome is the quantity and frequency of alcohol consumption in terms of standard drinks (10 g ethanol per standard drink) per month; this will be assessed through the timeline follow-back (TLFB) method [44], which measures alcohol consumption during the prior 30 days in terms of number of standard drinks. The TLFB is widely used in obtaining data on the frequency of occurrence of drinking behavior, has been evaluated in various clinical and nonclinical populations, and has been shown to have good psychometric characteristics [45,46].

#### Secondary Outcomes

##### Intervention Adherence

Intervention adherence will be calculated descriptively based on the percentage of participants who completed the main PNF and motivational interviewing module.

##### Readiness to Change

The Readiness to Change Questionnaire (RCQ) [47] is a 12-item self-report questionnaire assessing participants’ readiness to change according to the three stages of change: precontemplation, contemplation, and action. The items are rated on a 5-point scale from –2 (totally disagree) to 2 (totally agree). The scores of each stage of change are calculated by adding the individual item scores of the relevant scale. The range of each scale is –8 through 0 to +8. A negative score indicates an overall disagreement with the items measuring the particular stage of change, whereas a positive score represents overall agreement. The RCQ has previously been applied in Dutch clinical settings and has proven to be a reliable and valid tool to measure an individual’s readiness to change [48,49].

##### Binge Drinking

Participants will be asked to indicate the number of binge-drinking days defined as 4 (for females) or 5 (for males) standard drinks on one drinking occasion over the past 30 days.

##### Alcohol-Related Problem Severity

Alcohol-related problem severity will be assessed via the Rutgers Alcohol Problem Index (RAPI) [50]. The RAPI is an 18-item self-reporting scale that measures alcohol-related problems. The questions are answered on a 4-level scoring system, with higher scores indicating higher levels of alcohol-related problems. The total score is derived by summing the individual scores for each of the 18 items on a scale of 0 to 3; consequently, total scores range from 0 to 54. The RAPI is widely used to assess alcohol-related problems in adolescents and university students and is considered reliable and valid [50].

##### Cannabis Use Frequency and Quantity

Cannabis use frequency was assessed using the TLFB method [51]. Furthermore, we asked participants to estimate the quantity of cannabis consumed (in grams) during the previous 30 days. The TLFB method has shown to collect reliable and psychometrically sound information about individuals’ daily cannabis use [52].

##### Depressive Symptoms

Depressive symptoms will be assessed via the Center for Epidemiological Studies Depression (CES-D) scale [53], a 20-item self-report scale designed to measure depressive symptoms. The CES-D has been tested in various settings and is considered a reliable and valid tool to measure depressive symptoms in a variety of populations also including young adults [54]. Responses are based on the frequency of occurrence of depressive symptoms. The questionnaire uses a 4-point ordinal scale: 0 (not at all), 1 (several days), 2 (more than half the days), and 3 (nearly every day). The scores of the CES-D scale range between 0 and 60, with cut-off scores of 16 and 20 indicative of mild depressive symptoms, scores between 21 and 25 are indicative of moderate depressive symptoms, and scores between 26 and 60 suggest severe depressive symptoms [55].

##### Mental Well-being

The Warwick-Edinburgh Mental Wellbeing Scale (WEMWBS) [56] is a 14-item self-report scale that measures positive mental well-being. The items are rated on a scale of 1 (none of the time) to 5 (all of the time). Higher scores indicate higher levels of positive mental well-being among respondents. The WEMWBS has been previously validated in a Dutch setting and has proven to be a reliable and valid tool to measure individuals’ mental well-being [57].

##### Perceived Stress

The Perceived Stress Scale (PSS) [58] is a 10-item self-report scale that measures perceived stress. The questions are answered on a 5-level scoring system in which higher scores indicate higher levels of perceived stress. The total score is derived by summing the individual scores for each of the 10 items on a scale of 0 to 4; consequently, total scores range from 0 to 40. The PSS is considered a reliable and valid screening tool for perceived stress [59].

##### Study or Work Performance

The task performance scale is a 5-item self-report scale that measures study or work performance and is part of the Individual Work Performance Questionnaire [60]. The total score is derived by summing the individual scores for each of the 5 items on a scale of 0 to 4; consequently, total scores range from 0 to 20. The task performance scale is considered a reliable and valid screening tool for measuring study or work performance among adults [61].

##### Engagement

Engagement will be measured via the Twente Engagement with eHealth Technologies Scale (TWEETS) [62], a 9-item self-report scale that can be used to measure expectations of current or past engagement with digital interventions. In this study, the expected and past engagement will be assessed. The questions are answered on a 5-level scoring system in which higher scores indicate higher levels of treatment engagement. The total score is derived by summing the individual scores for each of the 9 items on a scale of 0 to 4; consequently, total scores range from 0 to 40. The TWEETS has shown to be a valid tool that possesses good psychometric qualities to assess engagement with eHealth technologies [63].

##### Short-Form Posttraumatic Stress Disorder Checklist for Diagnostic and Statistical Manual of Mental Disorders, 5th Edition

The short-form version of the posttraumatic stress disorder (PTSD) Checklist for Diagnostic and Statistical Manual of Mental Disorders, 5th Edition (DSM-5), or PCL-5 [64], is a 4-item self-report scale that screens for PTSD symptoms according to the DSM-5. The items are rated on a scale of 0 (not at all) to 4 (extremely). Higher scores indicate a higher likelihood for the screened individuals to fulfill the criteria of PTSD according to the DSM-5. However, the PCL-5 is meant to provide a provisional PTSD diagnosis in circumstances where a structured clinical interview is not feasible. The short-form PCL-5 has been previously validated in the Dutch population and has proven to be a reliable and valid tool [65].

##### Short-Form PCL-5 – COVID-19

Given the unprecedented situation that emerged due to the global COVID-19 pandemic, we decided to include a modified version of the short-form PCL-5 that measures only stressors tied to the COVID-19 pandemic. The short-form PCL-5 – COVID-19 scale that we used for this study is identical to the previously introduced short-form PCL-5; however, we specifically asked participants to indicate on this scale only stressors that are associated with the COVID-19 pandemic.

##### Urgency, Premeditation, Perseverance, Sensation Seeking, Positive Urgency Impulsive Behavior Scale

The reason we decided to include this measurement tool is due to the fact that impulsivity is a significant risk factor for the initiation, continuation, and problematic alcohol consumption [66]. Impulsivity will be assessed via the short-form Urgency, Premeditation, Perseverance, Sensation Seeking, Positive Urgency (UPPS-P) Impulsive Behavior Scale [67]. This short-form version of the UPPS-P is a 20-item self-report scale designed to measure factors that can lead to impulsive behaviors. The questionnaire items are scored on a scale of 1 (strongly disagree) to 4 (strongly agree). The UPPS-P contains five subscales, namely, positive urgency, negative urgency, (lack of) premeditation, (lack of) perseverance, and sensation seeking. The UPPS-P has been tested in healthy, student, and forensic psychiatry settings involving Dutch participants and is considered a reliable and valid tool [68].

#### Sociodemographic Variables

At baseline, sociodemographic variables such as sex, age, level of education, employment, and marital status will be assessed.

### Statistical Analyses

After the data collection period, we will clean our data and assess it for accuracy and completeness. Missing data will be handled using either multiple regression imputation techniques or full information maximum likelihood estimation, depending on the amount of missing data and whether it is missing at random or not. All analyses for the primary and secondary outcomes will use linear mixed modeling regression analyses with the three time points nested within participants. For continuous outcomes, linear models will be used for normally distributed data and negative binomial models, for left-skewed data. For dichotomous outcomes, such as whether or not participants had a binge-drinking episode in the past 30 days, binary logistic regression will be used. All primary analyses will be conducted in line with the intention-to-treat (ITT) principle (ie, including all participants that are randomized). Sensitivity analyses will be conducted in line with the per-protocol principle (ie, intervention completers) and with study completers only. All statistical tests use a significance level α=.05. Time will be used as predictor and baseline measures will be included as covariates if appropriate. All analyses will be conducted using the R Statistical Package. Cohen *d* values will be used as the measure of effect size. Effect sizes of 0.8 are assumed to be large, effect sizes of 0.5 are assumed to be moderate, and effect sizes of 0.2 are assumed to be small [69]. In addition, several intervention effect moderators that might moderate drinking levels based on findings of previous studies will be investigated. In addition, potential moderators such as gender [70], readiness to change [71], engagement [72], and impulsivity [73] will be explored.

## Results

Our study began recruitment in September 2020. We received 933 registrations on our study information website. Of these registrations, 506 participants have completed the baseline assessment, 336 participants have completed the 6-week postbaseline assessment (T1), and 308 participants have completed the 3-month postbaseline assessment (T2) as of May 2021. The study is still in progress. Results will be reported in 2021 and 2022.

## Discussion

### Study Overview

The majority of digital interventions aimed at reducing drinking levels in young adults were conducted in university settings in the United States or Australia, were delivered via a computer or web browser, and consisted of single-session interventions showing small effect sizes ranging from g=0.18 to g=0.29 [5,14-18]. Mobile apps with the purpose of managing alcohol consumption in young adults seem promising, but the evidence is still inconclusive given the mixed results [13].

To potentially enhance the effectiveness of such interventions, we decided to develop a self-guided mobile app supporting low-risk drinking habits among young adults from the general population, while incorporating relevant healthy lifestyle-related support components that may increase and prolong the effectiveness of our intervention but retain the acceptability and reach of single-session interventions [22-24].

Therefore, the present study aims at improving the existing knowledge on the effectiveness of mobile apps on alcohol reduction in young adults by employing lifestyle-related support components (ie, sleep, mood, and perceived stress). If our intervention proves to be effective, it could become an inexpensive and scalable public health intervention to improve drinking habits in young adults.

### Potential Strengths and Limitations

Our study has a number of strengths such as the delivery of our intervention via the use of a mobile app, which seems to be a feasible and desirable delivery method for our target group [74,75]. Second, this study used an open recruitment strategy, which will enhance the generalizability of our results compared to studies that recruit only in university settings. Given the large sample size we aim to recruit, our study will provide a reliable estimate regarding the effectiveness of our mobile intervention.

Nevertheless, there are some limitations that are worthwhile mentioning. We are measuring all outcomes via self-report measures, and we have decided against using lengthy and costly diagnostic interviews in order to reach out to a large sample group and due to the preventive nature of our intervention. However, based on the literature, we know that self-report measures are reliable for actual drinking levels in our target population [76]; furthermore, we expect that the EMA function that we included for self-monitoring purposes will augment and improve our assessments by adding a dynamic dimension to it [41]. We expect a potential large dropout rate at posttest and 3-month follow-up assessments as commonly observed in web-based interventions with an open recruitment strategy [77]. To minimize drop-out, automated reminders for completing questionnaires will be sent via email to our participants and participants will be offered financial incentives for completing the follow-up assessments.

### Conclusions

Providing young adults with a mobile app that promotes low-risk drinking habits while also providing lifestyle-related support components might be a promising approach to increase the reach, uptake, and effectiveness of behavioral change interventions targeting alcohol reduction in this population. We expect that the use of our mobile app will reduce drinking outcomes significantly compared to the use of the educational website. In addition, findings of the trial will be informative for future researchers developing interventions for young adults with regard to the reduction of alcohol consumption and improvement of associated lifestyle behaviors.

## Appendix

Multimedia Appendix 1 CONSORT-eHEALTH checklist (V 1.6.1).

## Abbreviations

CES-D: Center for Epidemiological Studies Depression
EDC: Electronic Data Capture
DSM-5: Diagnostic and Statistical Manual of Mental Disorders, 5th Edition
EMA: ecological momentary assessments
ITT: intention-to-treat
ORBITAL: Outcome Reporting in Brief Intervention Trials on Alcohol
PCL-5: Posttraumatic Stress Disorder Checklist for Diagnostic and Statistical Manual of Mental Disorders, 5th Edition
PNF: personalized normative feedback PSS: Perceived Stress Scale
PTSD: posttraumatic stress disorder
PSS: Perceived Stress Scale
RAPI: Rutgers Alcohol Problem Index
RCT: randomized controlled trial
RCQ: Readiness to Change Questionnaire
TLFB: timeline follow-back
TWEETS: Twente Engagement with eHealth Technologies Scale
UPPS-P: Urgency, Premeditation, Perseverance, Sensation Seeking, Positive Urgency
WEMWBS: Warwick-Edinburgh Mental Wellbeing Scale
